# Postsurgical *Pantoea calida* meningitis: a case report

**DOI:** 10.1186/1752-1947-8-195

**Published:** 2014-06-16

**Authors:** Shirley Fritz, Nadim Cassir, Remy Noudel, Silvestre De La Rosa, Pierre-Hugues Roche, Michel Drancourt

**Affiliations:** 1 URMITE, UMR63 CNRS 7278, IRD 198, Inserm 1095, Aix Marseille Université, 13005 Marseille, France; 2 Service des Maladies Infectieuses et Tropicales, Hôpital Nord, Assistance Publique – Hôpitaux de Marseille, Chemin des Bourrely, 13915 Marseille Cedex 20, France; 3 Service de Neurochirurgie, Hôpital Nord, Assistance Publique – Hôpitaux de Marseille, Chemin des Bourrely, 13915 Marseille Cedex 20, France; 4 Faculté de Médecine, Unité de recherche sur les maladies infectieuses et tropicales émergentes, 27, Boulevard Jean Moulin, Marseille Cedex 5, France

**Keywords:** Health-care associated infection, Meningitis, Neurosurgery, *Pantoea calida*

## Abstract

**Introduction:**

*Pantoea calida,* a recently described environmental Enterobacteriaceae organism, has not yet been associated with human infection.

**Case presentation:**

We report a case of postoperative meningitis caused by *P. calida.* After pituitary adenoma resection, a 52-year-old Caucasian woman developed febrile meningitis confirmed by cerebrospinal fluid analysis. *P. calida* was grown in pure culture from this fluid and was firmly identified with partial *rpoB* gene sequencing. She was cured by a 14-day course of meropenem.

**Conclusions:**

*P. calida* must be added to the list of opportunistic Enterobacteriaceae pathogens responsible for postsurgical meningitis. It is easily identified by matrix-assisted laser desorption/ionization time-of-flight mass spectrometry.

## Introduction

*Pantoea calida* has been recently described as a species of Enterobacteriaceae after its seminal isolation and culture from powdered infant formula [1]. It has not yet been associated with any infections and no clinical isolate has been reported. Here, we isolated one strain of *P. calida* from a cerebrospinal fluid (CSF) specimen and identified it as the cause of postsurgical meningitis.

## Case presentation

A 52-year-old Caucasian woman had a medical history of hypertension, smoking and alcoholism. She presented with binocular diplopia. A computed tomography scan showed an intrasellar lesion with invasion of her right cavernous sinus that was further confirmed by brain magnetic resonance imaging. An endoscopic endonasal biopsy was performed and histopathology showed a nonspecific inflammation and subacute hemorrhagic alteration of her nasal mucosa. An antibiotic prophylaxis with cefazolin 2gr was given. A transnasal surgical excision was performed, and no leak of CSF was observed after the surgery. Histopathology confirmed a single prolactin pituitary adenoma. Five days after the surgery, she presented a fever (38.6°C) and a meningeal syndrome with consciousness disorder, neck stiffness and a Glasgow Coma Scale evaluated at 13 (E4V4M5) but without focal neurological deficits. The CSF collected by lumbar puncture showed hypoglycorrhachia (1.33mmol/L; CSF/serum glucose ratio=0.20), an elevated protein level (5.88g/L) and 4500 leukocytes/mL including 98% polymorphonuclear leukocytes. There was no detectable organism on direct microscopic examination after Gram staining. Treatment combining intravenous vancomycin 1gr twice a day and intravenous meropenem 2gr three times a day was started for presumptive postsurgical meningitis. Her clinical condition rapidly improved, with apyrexia and a net regression of the meningeal syndrome within 2 days. Five days after the onset of symptoms, vancomycin was stopped and meropenem 6gr per day was continued for a total of 14 days. At 90-day follow-up she was afebrile, healthy and without any sign of meningitis.

The CSF was inoculated on Polyvitex (bioMérieux, La Balme-les-Grottes, France) and Columbia agar with 5% sheep blood and incubated at 37°C under a 5% carbon dioxide atmosphere. After 48-hour incubation, small whitish round shiny domed colonies were observed on both culture media. Matrix-assisted laser desorption/ionization time-of-flight mass spectrometry (MALDI-TOF-MS) performed as previously described [2,3] yielded *P. calida* with identification score of 2.26, 2.17 and 2.17 for the three spots deposited on the MALDI-TOF-MS plate. Furthermore partial *rpoB* gene sequencing performed as previously reported [4] confirmed the identification of *P. calida* with 99.88% sequence similarity with the reference sequence (GenBank GQ892191). Antimicrobial susceptibility was performed by diffusion method in agar and incubated aerobically for 24 hours at 37°C. The isolate was resistant to ampicillin (minimal inhibitory concentration (MIC) >8mg/L), amoxicillin-clavulanate (MIC, 1mg/L), and cephalothin (MIC, 1mg/L). It was susceptible to ceftriaxone (MIC, 0.5mg/L), imipenem (MIC, 0.5mg/L), ciprofloxacin (MIC, 0.25mg/L), gentamicin (MIC<1mg/L) and co-trimoxazole (MIC<1mg/L).

## Discussion

Here, *P. calida* was isolated from the CSF of a patient with postoperative meningitis [5]. The isolate was firmly identified by using two different complementary approaches: MALDI-TOF-MS which is an advanced proteomics method [2,3] relying on the analysis of unique peptidic signatures; and partial *rpoB* gene sequencing which relies on the analysis of unique nucleotidic signatures [6]. These two technical approaches yielded unambiguous, concordant identification. In particular, MALDI-TOF-MS proved to be efficient to identify this species, despite the fact that no clinical isolate was previously included in the database.

The absence of any bacteria other than *P. calida* on the culture of CSF, as well as the fact that it was the first identification of this microorganism in our laboratory, secure the conclusion that *P. calida* was responsible for this postoperative meningitis. Accordingly, the patient was rapidly cured after an effective antibiotic treatment was administrated. In this case, the source of infection remained unknown. Indeed, *P. calida* has been documented only in powdered infant formula [1] and our patient had no contact with such formula. Of interest, the *Cronobacter* species (formerly *Enterobacter sakazakii*) causing health-care-associated meningitis in neonates [7] is also contaminating powdered infant formula [8,9].

Indeed, *P. calida* is closely related to members of the Enterobacteriaceae genus *Erwinia*, *Tatumella*, *Kluyvera*, *Citrobacter* and *Cronobacter*, which also comprise opportunistic pathogens causing meningitis, mainly in newborns [1]. Whereas *Erwinia* and *Tatumella* organisms have not been associated with central nervous system infection, *Kluyvera* meningitis has been reported in a newborn [10]. Also *Citrobacter* bacteria are opportunistic pathogens seldom reported as causing meningitis in neonates [11], children [12] and adults with cancer [13]. Recently, one case of *Citrobacter koseri* meningitis has been reported after free-diving [14]. Lastly, *Cronobacter* species along with *Enterobacter* species have been reported as causes of health-care-associated meningitis [15].

## Conclusions

*P. calida* should be added to the list of Enterobacteriaceae pathogens responsible for infectious postoperative meningitis. Sources different from powdered infant formula should be investigated. *P. calida* meningitis can be cured by appropriate antibiotic treatment. This new pathogen can easily be identified by using MALDI-TOF-MS [2,3].

## Consent

Written informed consent was obtained from the patient for publication of this case report and accompanying images. A copy of the written consent is available for review by the Editor-in-Chief of this journal.

## Abbreviations

CSF: Cerebrospinal fluid; MALDI-TOF-MS: Matrix-assisted laser desorption/ionization time-of-flight mass spectrometry; MIC: Minimal inhibitory concentration.

## Competing interests

The authors declare that they have no competing interests.

## Authors’ contributions

NC, RN, SDLR, and PHR took care of the patient. SF and MD took care of the microbiology of the specimens. SF, NC, RN, and SDLR wrote the case. PHR and MD evaluated the draft and suggested revisions. All authors read and approved the final manuscript.

## References

[B1] Popp A Cleenwerck I Iversen C De Vos P Stephan R *Pantoea gaviniae* sp. nov. and *Pantoea calida* sp. nov., isolated from infant formula and an infant formula production environment Int J Syst Evol Microbiol 2010 60 2786 2792 10.1099/ijs.0.019430-0 20061487

[B2] Seng P Drancourt M Gouriet F La Scola B Fournier PE Rolain JM Raoult D Ongoing revolution in bacteriology: routine identification of bacteria by matrix-assisted laser desorption ionization time-of-flight mass spectrometry Clin Infect Dis 2009 49 543 551 10.1086/600885 19583519

[B3] Seng P Abat C Rolain JM Colson P Lagier JC Gouriet F Fournier PE Drancourt M La Scola B Raoult D Identification of rare pathogenic bacteria in a clinical microbiology laboratory: impact of matrix-assisted laser desorption ionization-time of flight mass spectrometry J Clin Microbiol 2013 51 2182 2194 10.1128/JCM.00492-13 23637301 PMC3697718

[B4] Mollet C Drancourt M Raoult D *rpoB* sequence analysis as a novel basis for bacterial identification Mol Microbiol 1997 26 1005 1011 10.1046/j.1365-2958.1997.6382009.x 9426137

[B5] Tavares WM Machado AG Matushita H Plese JP CSF markers for diagnosis of bacterial meningitis in postoperative neurosurgical patients Arq Neuropsiquiatr 2006 64 592 595 10.1590/S0004-282X2006000400012 17119799

[B6] Adékambi T Drancourt M Raoult D The *rpoB* gene as a tool for clinical microbiologists Trends Microbiol 2009 17 37 45 10.1016/j.tim.2008.09.008 19081723

[B7] Hunter CJ Bean JF *Cronobacter*: an emerging opportunistic pathogen associated with neonatal meningitis, sepsis and necrotizing enterocolitis J Perinatol 2013 33 581 585 10.1038/jp.2013.26 23538645

[B8] Cai XQ Yu HQ Ruan ZX Yang LL Bai JS Qiu DY Jian ZH Xiao YQ Yang JY Le TH Zhu XQ Rapid detection and simultaneous genotyping of *Cronobacter* spp. (formerly *Enterobacter sakazakii*) in powdered infant formula using real-time PCR and high resolution melting (HRM) analysis PLoS One 2013 8 6 e67082 10.1371/journal.pone.0067082 23825624 PMC3692429

[B9] Yan QQ Condell O Power K Butler F Tall BD Fanning S *Cronobacter* species (formerly known as *Enterobacter sakazakii*) in powdered infant formula: a review of our current understanding of the biology of this bacterium J Appl Microbiol 2012 113 1 15 10.1111/j.1365-2672.2012.05281.x 22420458

[B10] Rosso M Rojas P Garcia E Marquez J Losada A Munoz M *Kluyvera* meningitis in a newborn Pediatr Infect Dis J 2007 26 1070 1071 10.1097/INF.0b013e31812e4b96 17984822

[B11] Cuadros EN Castilla CY Algarra CM Pérez DM Lopez BR Martin FJ Cardona AU Medical and neurosurgical management of *Citrobacter koseri,* a rare cause of neonatal meningitis J Med Microbiol 2014 63 144 147 10.1099/jmm.0.063586-0 24243285

[B12] Prais D Nussinovitch M Harel L Amir J *Citrobacter koseri* (*diversus*) meningitis in an otherwise healthy adolescent Scand J Infect Dis 2003 35 202 204 10.1080/0036554021000027020 12751719

[B13] Tan CK Lai CC Lin SH Liao CH Huang YT Hsueh PR Fatal *Citrobacter farmeri* meningitis in a patient with nasopharyngeal cancer J Clin Microbiol 2010 48 1499 1500 10.1128/JCM.00282-10 20181904 PMC2849549

[B14] Pollara G Savy L Cropley I Hopkins S *Citrobacter koseri* meningitis: another freediving risk? J Infect 2011 62 101 103 10.1016/j.jinf.2010.09.036 20933000

[B15] Kuo CC Wang JY Chien JY Chen YF Wu VC Tsai CW Hwang JJ Nontraumatic pneumocephalus due to nosocomial *Enterobacter cloacae* infection Diagn Microbiol Infect Dis 2010 66 108 110 10.1016/j.diagmicrobio.2009.03.024 19442474

